# Giant umbilical hernia: “Lazy M-Omega” flap, a case report

**DOI:** 10.11604/pamj.2021.38.338.23255

**Published:** 2021-04-07

**Authors:** Vanesa Villamil, Oscar Girón Vallejo, Juana María Sánchez Morote, Juan Pedro Hernández Bermejo

**Affiliations:** 1 Pediatric Surgery Service, Sant Joan de Déu Children's Hospital, Barcelona, Spain,; 2 Pediatric Surgery Service, University Clinical Hospital Virgen de la Arrixaca, Murcia, Spain

**Keywords:** Umbilical hernia, umbilicus, reconstruction, case report

## Abstract

Umbilical hernia, one of the most frequent pathologies in pediatric surgical practice, is usually corrected with a relatively simple intervention, except in cases where there is a major defect, also called proboscoid hernia. We present a case report of a 20-month male patient that underwent surgical intervention of a giant umbilical hernia with the “Lazy-M and Omega” flap novel surgical technique. This technique has to be taken into account in surgical planning, since it is simple and easily reproducible.

## Introduction

Umbilical hernia, one of the most frequent pathologies in pediatric surgical practice, is usually corrected with a relatively simple procedure, except in cases where there is a major defect, or also called proboscoid hernia [1]. Several techniques have been proposed in order to address this problem [1, 2]. In our case, the technique we opted for was the “Lazy M-Omega” flap, published in 2004 by Tamir *et al*. [3]. We present the clinical case of a male patient of 20 months of life to whom an intervention of umbilical hernia is performed due to the size of it.

## Patient and observation

A patient with no personal or family history of interest, is seen in consultations from birth due to a large umbilical hernia. Physical examination revealed a soft abdomen, depressible, not painful, without masses or visceromegaly, with an umbilical hernial defect of approximately 3 cm in diameter and an excess of skin of approximately 5 cm in length. After a year and a half of follow-up and seeing that the umbilical hernia was increasing in size and due to the large excess of soft tissue, we decided to include him on the waiting list for surgical correction of the hernia.

**Figure 1:**
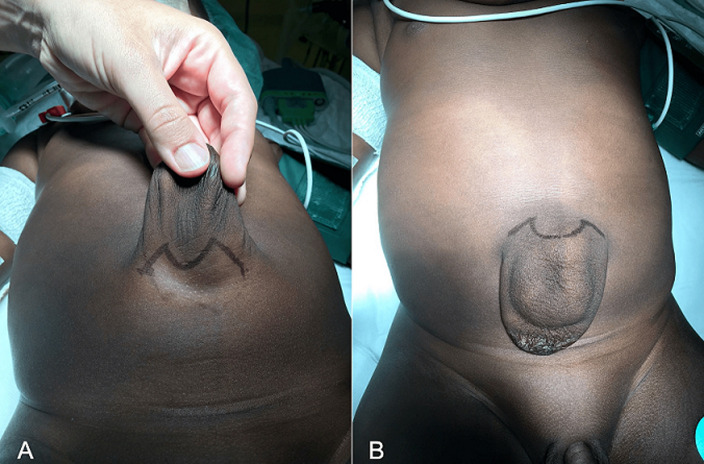
A) lower incision in “Lazy M”; B) superior incision in “Ω” (omega)

**Figure 2:**
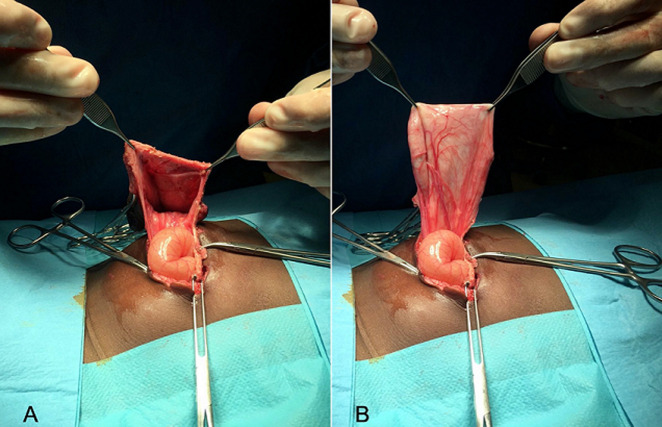
great hernial sac; A) inverted; B) everted

**Figure 3:**
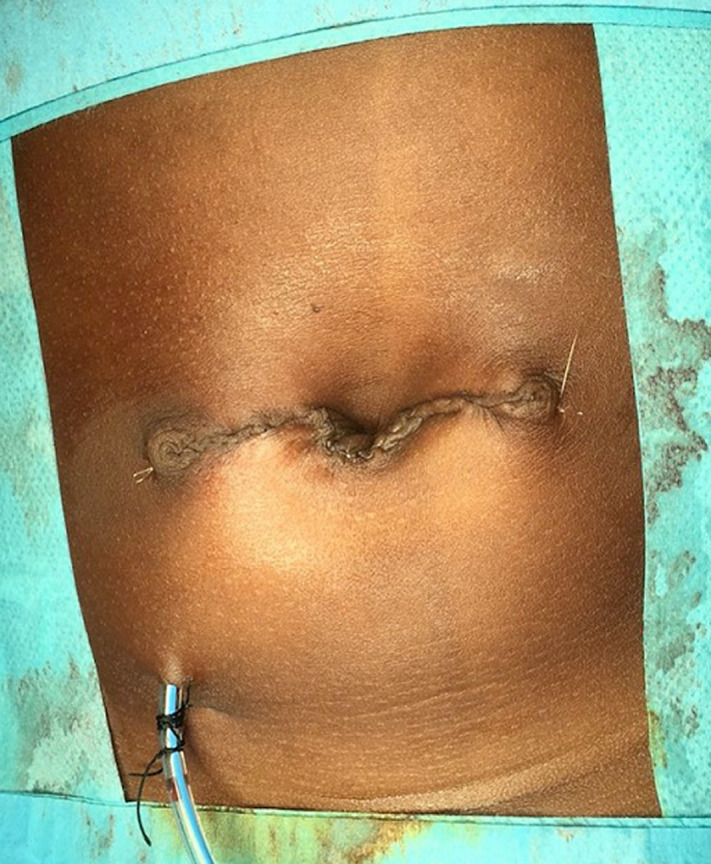
postoperative appearance

At 20 months of age, the patient underwent surgery. Already on the operating table, two letters are drawn on the leftover skin. An open “M” through the infraumbilical area (Figure 1 A) and then an inverted omega above the umbilicus (Figure 1 B), which would assume the negative shape of the letter “M”. With these two incisions, an opening of the hernial sac and a dissection thereof is performed until the healthy edges of the aponeurosis are those that will be used to repair the defect. After resecting the large hernial sac (Figure 2 A and Figure 2 B), performing the herniorrhaphy and removing the excess skin, the two edges are sutured, being perfectly balanced; the convexity of the “Ω” will conform the new umbilicus (Figure 3). Due to the large dissection of the tissues, it was decided to leave a drain for 24 hours. In the immediate postoperative period, the patient began breastfeeding without problems and 12 hours after surgery, he began to have stools. The day after surgery, once the drain was removed, the patient was discharged.

## Discussion

The umbilical hernia is a common problem in children. A large one often requires umbilicoplasty for a protruding umbilicus with redundant skin. The proboscoid variety of umbilical hernia protrudes through the fascial defect [4]. For most children, umbilical hernias often close spontaneously and can be monitored safely through watchful waiting. Despite the fact that in the surgical calendar umbilical hernias must be operated on at 4 years of age, some surgeons have advocated a practice of repairing “giant” umbilical hernias earlier than smaller hernias, like we did in this particular case. One reason for this practice may be because larger defects are less likely to close spontaneously [5]. Due to the high frequency of umbilical hernias in the pediatric population, many of which require surgical repair, multiple factors should be considered when determining the appropriate surgical approach [6]. Treatment of proboscoid umbilical hernia in children includes two steps: umbilical ring closure and aesthetic reconstruction of the umbilicus. The aim of the surgeon in the surgical correction of umbilical hernia, is to create an umbilicus of natural appearance, which consists of a ring, a tubular wall, a sulcus and a bottom without any excess skin to preserve the aesthetic aspect of the umbilicus [7]. Several techniques have been described on the surgical correction of it [1, 2, 4, 8], but none adapted to the magnitude of excess skin that our patient had, except for the technique proposed by Tamir *et al*. [3]. In this case, the problem is not in the aponeurosis closure but the removal of the excess skin and achieving a satisfactory aesthetic effect. The Lazy-M and inverted Omega flaps described here offer a simple, easy-to-plan and easy-to-use technique with predictable aesthetic result. Written consent for publication was obtained from the patient or their relative.

## Conclusion

In cases such as the one presented with a giant umbilical hernia, the “Lazy-M and Omega” flap is a technique to be taken into account in surgical planning, since it is simple and easily reproducible.

## References

[1] Reyna TM, Hollis HW, Smith SB (1987). Surgical management of proboscoid herniae. J Pediatr Surg.

[2] Sankalé A, Ngom G, Fall I, Coulibaly NF, Ndoye M (2004). Umbilical reconstruction in children: prospective report of 77 cases. Ann Chir Plast Esthet.

[3] Tamir G, Kurzbart E (2004). Umbilical reconstruction after repair of large umbilical hernia: The “Lazy-M” and omega flaps. J Pediatr Surg.

[4] Bawazir OA (2019). A new umbilicoplasty technique for the management of large umbilical hernia in children. Hernia.

[5] Zens TJ, Cartmill R, Muldowney BL, Fernandes-Taylor S, Nichol P, Kohler JE (2019). Practice variation in umbilical hernia repair demonstrates a need for best practice guidelines. J Pediatr.

[6] Pallister ZS, Angotti LM, Patel VK, Pimpalwar AP (2019). Transumbilical repair of umbilical hernia in children: the covert scar approach. J Pediatr Surg.

[7] El-Dessouki NI, Shehata SMK, Torki AM, Hashish AA (2004). Double half-cone flap umbilicoplasty: a new technique for the proboscoid umbilical hernia in children. Hernia.

[8] Ashu EE, Leroy GM, Aristide BG, Joss BML, Bonaventure J, Patrick SE (2015). Double half-cone flap umbilicoplasty for proboscoid umbilical hernia in a 2 years old child with satisfactory results 2 years later. Pan Afr Med J.

